# Vitamin D Is a Good Marker for Disease Activity of Rheumatoid Arthritis Disease

**DOI:** 10.1155/2015/260725

**Published:** 2015-05-10

**Authors:** Firas Sultan Azzeh, Osama Adnan Kensara

**Affiliations:** Department of Clinical Nutrition, Faculty of Applied Medical Sciences, Umm Al-Qura University, Makkah 21955, Saudi Arabia

## Abstract

*Aim*. This study was conducted to find out the optimal vitamin D cutoff point in predicting activity of RA disease. *Materials and Methods*. One hundred and two rheumatoid arthritis Saudi patients of both genders were recruited in this study. Vitamin D as 25-hydroxy-vitamin D [25(OH)D] was measured and serum level less than 20 ng/mL defined as deficient patient. Disease activity was measured based on the disease activity score index of a 28-joint count (DAS28) using serum erythrocyte sedimentation rate levels. Receiver operating characteristic (ROC) curves were used to determine the optimal vitamin D cutoff points for identifying disease activity. *Results*. It has been observed that vitamin D levels were lower (*P* < 0.05) in patients with high disease activity. A significant inverse correlation between serum 25(OH)D levels and DAS28 (*r* = −0.277, *P* = 0.014) was shown. ROC curves results showed that vitamin D less than 12.3 ng/mL predicted high disease activity, and vitamin D more than 17.9 ng/mL predicted low disease activity, with good sensitivity and accuracy results regarding vitamin D. *Conclusion*. Study results concluded that vitamin D is a good predictor of RA disease activity in Saudi patients.

## 1. Introduction

Rheumatoid arthritis (RA) is one of the autoimmune diseases that caused inflammation of the synovium with continuous erosion of bone leading to loss of the joint [1]. The etiology of the disease could be attributable to genetic and nongenetic factors as hormonal, environmental, and infectious factors [2]. Vitamin D could be one of the environmental factors related to RA disease [3]. The immunomodulatory effects of vitamin D and the detection of vitamin D receptors in the immune system cells may elucidate the associations between the vitamin and RA [4, 5]. Vitamin D was postulated by experts for its role in cellular proliferation and differentiation and survival of cells in immunity disorders [6]. Also, production of vitamin D hormone after immune dendritic cells activation may suggest that vitamin D could have immune-regulatory properties [2, 7].

Vitamin D deficiency was found to be common in RA patients [5, 8–10], and an inverse correlation existed in serum vitamin D levels and RA activity [7–12]. Previous outcomes were also achieved in Saudi Arabia as published by Attar [13] and Atwa et al. [14]. In addition, vitamin D intake was proved to have negative correlation with RA severity [15] and incidence [12].

Diagnostic procedures for RA disease could be performed by symptoms, antibodies, and inflammatory biomarkers, while genetic and environmental factors are more useful in early prognosis of the disease [16]. Blaney et al. [17] assumed that vitamin D could be used as clinical biomarker in RA disease and other autoimmune diseases. To the best of our knowledge, no detailed researches measure the optimal vitamin D cutoff point related to RA disease activity. Therefore, this study is novel on finding out the optimal vitamin D cutoff point in predicting activity of RA disease.

## 2. Subjects and Methods

### 2.1. Subjects

A cross-sectional study of a convenient sample of 102 patients out of a total population of 239 patients with RA of both genders and aged between 22 and 75 years were included in this study. Patients were recruited from Doctor Abdulrahman Taha Bakhsh Hospital (Jeddah, Western Saudi Arabia) during the study period from September 2012 to October 2013. Ethical approval was obtained from the University of Umm Al-Qura Ethics Committee. All recruited subjects signed the consent form before participating in this study.

**Figure 1:**
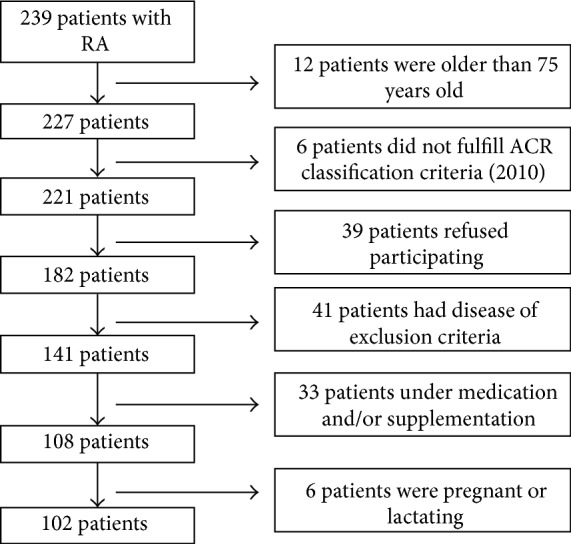
Study flow chart for recruiting RA patients.

RA classification criteria as defined by American College of Rheumatology and European League Against Rheumatism (ACR/EULAR) at 2010 [18] were adopted for diagnosis of RA patients. Exclusion criteria were as follows: older than 75 years old, mal-absorption, glomerular filtration rate less than 40 mL/min/1.73 m^2^, malignancy, diabetes, osteoporosis, systemic lupus erythematosus, hyperthyroidism, celiac disease, inflammatory bowel diseases, pregnancy, lactation, immobility for more than one week, current and/or long term usage of tuberculosis or fungal medications, receiving or have received hormonal replacement therapy and/or steroid medication, vitamin D supplementation before experiment, and having had bypass surgery.  Figure 1 shows the study flow chart for recruiting RA patients according to inclusion and exclusion criteria.

### 2.2. Methods

After having the signed consent form from participants, health status, body weight, and height were collected and the body mass index (BMI: Kg/m^2^) was calculated. Then blood samples (about 6 mL) were taken from each patient. Serums were obtained by centrifuged blood samples and quickly frozen and stored at −70°C until biochemical indicators were measured. The serum level of vitamin D as 25-hydroxy-vitamin D [25(OH)D] was measured using chemiluminescent immunoassay technique. Vitamin D defined normal if serum level was ≥30 ng/mL (≥75 nmol/L) and defined insufficient and deficient if serum level was between 20 and 30 ng/mL (50–75 nmol/L) and less than 20 ng/mL (<50 nmol/L) [19], respectively. Investigations including calcium (Ca: normal 2.12–2.52 mmol/L), phosphorus (Phos: normal 0.81–1.58 mmol/L), and alkaline phosphatase (ALP: normal 50–136 U/L) were done on ARCHITECT-ci16200 clinical chemistry analyzer instrument from Abbott Laboratories (Abbott Park, Illinois, USA). Erythrocyte sedimentation rate (ESR: normal 0–20 mm/hr) was measured by Westergren method. Hematology tests (complete blood count (CBC)) of white blood cells (WBC: normal 4.5–11.0 ×1000/*μ*L), hemoglobin (Hb: 11–16 g/dL), and platelets (PLT: 130–400 ×1000/*μ*L) were performed on COULTER GEN-S-SYSTEM-2 fully automated hematology analyzer instrument from Beckman coulter (2004, Florida, USA). Each parameter was completed according to a specific kit, and all previous instruments and methods were available at the laboratory department of Doctor Abdulrahman Taha Bakhsh Hospital.

Disease activity was measured based on the disease activity score index of a 28-joint count (DAS28) using serum ESR levels [20]. Consequently, patients were categorized into three groups by DAS28-ESR: (i) high disease activity (DAS28: >5.1), (ii) moderate disease activity (DAS28: 3.21–5.1), and (iii) low disease activity (DAS28: ≤3.2) [21].

### 2.3. Statistical Analysis

Statistical analysis was performed with SPSS software (Statistic Package for Social Sciences) version 20. Analysis of variance (ANOVA) test was used to compare significance between groups and post hoc test (least significance difference [LSD]) was used to compare significance within each variable. Bivariate Pearson correlation was carried out to study the correlation between the parameters. Multinomial logistic regression was used to calculate odds ratio (OR) and its 95% confidence intervals (95% CI) between vitamin D and disease activity (dependent variable). Receiver operating characteristic (ROC) curves were used to determine the optimal vitamin D cutoff points for identifying disease activity. Youden index (*J*) was used to determine the optimal cutoff points, as described by Akobeng [22]. *P* value less than 0.05 was considered statistically significant.

## 3. Results

Among the study sample, vitamin D deficiency was detected in 59 patients (57.8%) (25(OH)D <20 ng/mL), while 32 patients (31.4%) had vitamin D insufficiency (25(OH)D 20–30 ng/mL), and only 11 patients (10.8%) had vitamin D sufficiency (25(OH)D value ≥30 ng/mL). Regarding healthy status of the participants, only 24 patients (23.5%) were medically free from any of the chronic diseases. Hypertension (*n* = 28, 27.5%) was the most reported chronic disease in studied sample. Other chronic diseases were shown in low percentages as hyperuricemia (8.8%), fatty liver (4.9%), and hepatitis B (2%) and peptic ulcer, psoriasis, hyperlipidemia, sickle cell anemia, and fibromyalgia were shown in less than 1%.

Table 1 shows the baseline characteristics of the participants according to gender. The RA sample was mostly female (80.4%). The mean age of the whole sample was about 50 years and mean BMI for the whole study sample was 30.13 (kg/m^2^) indicating an obese sample. The mean vitamin D levels for male (17.46 ng/mL) and female (19.02 ng/mL) were within deficient category and no significant differences were found between them. Calcium, phosphorus, ALP, and CBC parameters all were observed at the normal ranges. The mean value of ESR showed to be greatly higher value than the normal range, and the mean score of DAS28-ESR for the entire sample was 3.79 providing a moderate disease activity group.

Table 2 demonstrates the baseline characteristics of the participants according to the disease activity. About 16.7% (*n* = 17) of the participants were classified as high disease activity, and around 54.9% (*n* = 56) and 28.4% (*n* = 29) were classified as moderate and low disease activity, respectively. There were no significant differences among the three groups with regard to age, height, weight, BMI, CBC, calcium, and PLT. The mean value of vitamin D decreased significantly (*P* = 0.023) as disease activity increased, and high disease activity subjects were lower (*P* ≤ 0.05) in their vitamin D as compared with the other two groups. However, the mean value of vitamin D level was 12.15 ng/mL in the high disease activity group, 20.63 ng/mL in the moderate disease activity group, and 23.41 ng/mL in the low disease activity group. Also, low disease activity group presented the lowest ESR and DAS28-ESR values. Phosphorus results exerted significant difference (*P* = 0.014) among all groups, but no clear tread was observed between groups.

**Figure 2:**
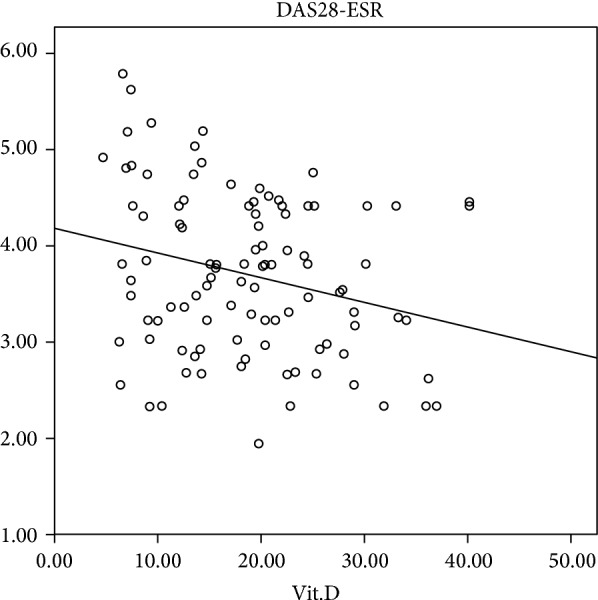
Correlation curve between vitamin D and DAS28-ESR activity scores.

Pearson correlation test revealed that there was a significant negative correlation between DAS28-ESR and vitamin D (*r* = −0.277, *P* = 0.014), as well as between DAS28-ESR and ESR (*r* = 0.453, *P* < 0.001) (Table 3).  Figure 2 demonstrates the correlation between vitamin D and DAS28-ESR (*r* square = 0.077, *P* = 0.014). The correlation between DAS28-ESR and vitamin D increased to −0.352 (*P* = 0.002) after age and gender was adjusted.

**Figure 3:**
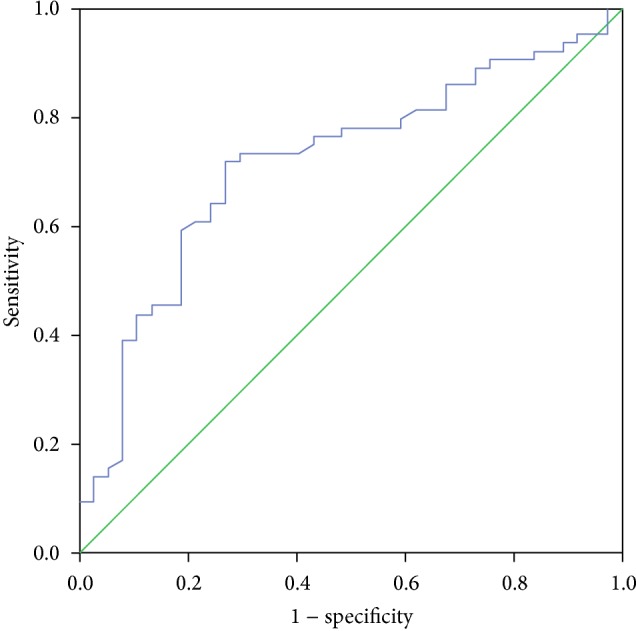
ROC curve for identifying optimal vitamin D cutoff point in predicting high disease activity (*J* = 12.3 ng/mL).

**Figure 4:**
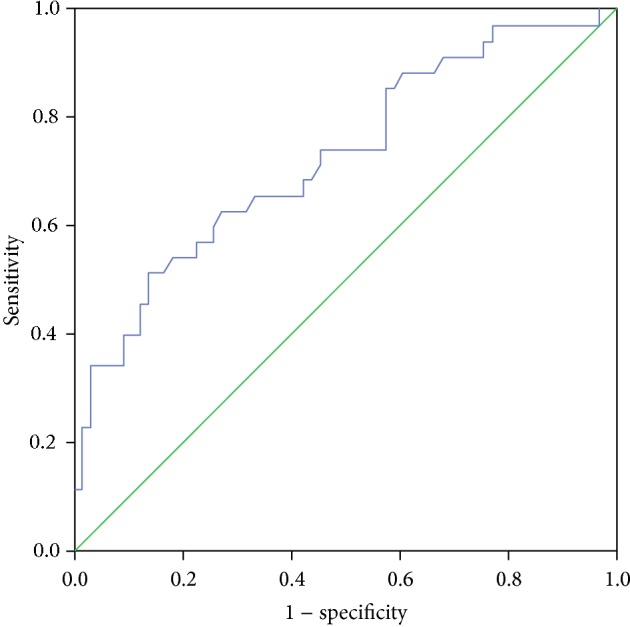
ROC curve for identifying optimal vitamin D cutoff point in predicting low disease activity (*J* = 17.9 ng/mL).

Figures 3 and 4 illustrate the ROC curves for identifying optimal vitamin D cutoff points in predicting high and low disease activity, respectively.  Figure 3 displays that area under the curve (AUC) was 0.716 (95% CI = 0.613–0.819, *P* value <0.001), and sensitivity and specificity were 82.6% and 75.4%, respectively. Optimal vitamin D cutoff point (*J*) in predicting high RA disease activity was 12.3 ng/mL (30.7 nmol/L). On the other hand, Figure 4 shows that AUC was 0.728 (95% CI = 0.622–0.834, *P* value <0.001), sensitivity was 78.4%, specificity was 70.9%, and optimal vitamin D cutoff point (*J*) was 17.9 ng/mL (44.7 nmol/L) in predicting low disease activity.

## 4. Discussion

Vitamin D as 25(OH)D was used in this study to determine the status of vitamin D because serum prohormone 25(OH)D with a half-life of 2 to 3 weeks was the best indicator of reflecting overall vitamin D status, since the half-life of 1,25-di-hydroxy-vitamin D [1,25(OH)_2_D] is only 3 to 4 hours, as reported by Khan and Fabian [23]. Activity of the disease was defined based on the DAS28-ESR, which is extensively validated and superior in interpretation of RA disease activity [21].

Study results showed that females were more susceptible to RA than males with ratio of about 4 : 1. Tobón et al. [1] was in consistence with last result which reported that the female-to-male RA ratio is 3 : 1, which could be related to differences in sex hormones. In addition, the fifth decade is the peak age for RA onset, which is a time of women hormonal alterations [24].

The relationship between vitamin D and RA was not totally recognized with inconsistent outcomes between researchers. Some case-control studies did not find differences in vitamin D status between RA patients and controls [7, 25, 26], and Feser et al. [26] and Craig et al. [27] investigations exerted that vitamin D had no effect on RA disease activity. Also, another cross-sectional study have found serum 1,25(OH)_2_D, but not 25(OH)D, to be inversely correlated with RA disease activity [28]. Low serum 25(OH)D was, however, associated with elevated RA disease activity in many researches [7–15]. Furthermore, the immunomodulatory effects of vitamin D could confirm this association [4–6]. Study results were in agreement with the existence of a relationship between vitamin D and RA disease activity by two effects: (1) vitamin D levels were lower (*P* < 0.05) in patients with high disease activity, with a clear trend toward a continuous increasing in 25(OH)D level along with decreasing disease activity; (2) also there was a significant inverse correlation between serum 25(OH)D levels and disease activity as evaluated by DAS28-ESR. Similar findings were shown in Saudi Arabia in which a significant negative correlation between vitamin D and disease activity of RA patients was obtained [13, 14]. In addition, multinomial logistic regression indicated that vitamin D deficient patients have about 26 (OR = 26.28, 95% CI: 8.55–74.13, *P* < 0.001) and 9 (OR = 9.44, 95% CI: 2.62–30.92, *P* = 0.004) times higher risk of developing high disease activity (DAS28-ESR) than patients with normal and insufficient vitamin D status, respectively. All these effects determined that vitamin D is a good marker of disease activity in Saudi RA patients. Blaney et al. [17] was in line with the previous result to the effect that vitamin D could be used as clinical biomarker in RA disease.

All published studies focused on finding the correlation between vitamin D and RA disease activity without determining the optimal cutoff points of vitamin D related to high or low disease activity. ROC curves (Figures 3 and 4) showed that the sensitivity (true positive) values in predicting high and low disease activity were 82.6% and 78.4%, respectively, which represents good results. Moreover, the AUC for both curves was more than 0.7 indicating good accuracy, since AUC between 0.7 and 0.9 represents moderate accuracy and more than 0.9 represents high accuracy [22]. Therefore, vitamin D is a good indicator as a predictor of RA disease activity. The best cutoff points of vitamin D that maximize sensitivity and specificity were 12.3 ng/mL (30.7 nmol/L) and 17.9 ng/mL (44.7 nmol/L) for high and low disease activity, respectively. These results demonstrate that as vitamin D in any Saudi RA patient was less than 12.3 ng/mL, it could be a strong predictor to have high disease activity. On the other hand, low disease activity could be predicted if vitamin D status was more than 17.9 ng/mL. So, disease activity of Saudi RA patients could be classified by DAS28-ESR and vitamin D status.

The study was limited by recruiting low male subjects, which limits us for having optimal vitamin D cutoff point by gender. We suggest further studies with large sample size to evaluate the predictive value of vitamin D with respect to gender as well as other disease activity measures as C-reactive protein.

## 5. Conclusion

This study concluded that vitamin D is a good predictor of RA disease activity in Saudi patients. The corresponding values of vitamin D for high disease activity (DAS28: >5.1) and low disease activity (DAS28: ≤3.2) are ≤12.3 ng/mL and ≥17.9 ng/mL, respectively.

## Figures and Tables

**Table 1:** Baseline characteristics of the participants according to gender.

Parameter	Male (*n* = 20)	Female (*n* = 82)	Total (*n* = 102)
Age (years)	53.72 ± 13.39	49.28 ± 10.29	50.09 ± 10.98
Height (cm)	169.12^a^ ± 8.04	156.96^b^ ± 7.65	159.22 ± 9.03
Weight (kg)	83.7^a^ ± 14.12	74.49^b^ ± 15.45	76.25 ± 15.56
BMI (kg/m^2^)	29.35 ± 4.18	30.31 ± 6.69	30.13 ± 6.29
WBC (×1000/*μ*L)	7.7 ± 2.5	7.16 ± 2.5	7.26 ± 2.5
Hb (g/dL)	12.79 ± 1.89	11.66 ± 1.46	11.87 ± 1.6
PLT (×1000/*μ*L)	276.79 ± 74.07	319.07 ± 98.95	311.04 ± 95.85
Vit.D (ng/mL)	17.46 ± 6.79	19.02 ± 8.7	18.73 ± 8.37
Ca (mmol/L)	2.32 ± 0.11	2.35 ± 0.85	2.35 ± 0.77
Phos (mmol/L)	1.31 ± 0.35	1.22 ± 0.32	1.23 ± 0.32
ALP (U/L)	124.12 ± 86.82	108.46 ± 66.84	111.55 ± 70.9
ESR (mm/hr)	48.27 ± 28.69	52.84 ± 23.1	52.02 ± 24.1
DAS28-ESR	3.72 ± 0.64	3.8 ± 0.92	3.79 ± 0.87

BMI: body mass index (18–25 kg/m^2^); WBC: white blood cells (4.5–11.0 ×1000/*μ*L); Hb: hemoglobin (12.0–16.0 g/dL); PLT: platelets (130–400 ×1000/*μ*L); Vit.D: vitamin D (sufficient >30 ng/mL), Ca: calcium (2.12–2.52 mmol/L); Phos: phosphorus (0.81–1.58 mmol/L); ALP: alkaline phosphatase (50–136 U/L); ESR: erythrocyte sedimentation rate (0–20 mm/hr); DAS28-ESR: disease activity score in 28 joints using erythrocyte sedimentation rate.

Letters with different superscripts in the same row are significantly (*P* < 0.05) different by *t*-test.

**Table 2:** Baseline characteristics of the participants according to the DAS28-ESR disease activity.

Parameter	High activity (*n* = 17)	Moderate activity (*n* = 56)	Low activity (*n* = 29)	*P* value
Age (years)	49.51 ± 5.13	50.97 ± 11.62	49.66 ± 8.92	0.655
Height (cm)	158.41 ± 6.83	160.66 ± 8.36	158.59 ± 11.29	0.728
Weight (kg)	75.41 ± 8.45	78.19 ± 14.78	72.10 ± 18.3	0.373
BMI (kg/m^2^)	30.51 ± 4.51	30.61 ± 5.51	29.32 ± 8.16	0.781
WBC (×1000/*μ*L)	7.41 ± 4	7.23 ± 2.28	7.03 ± 2.37	0.691
Hb (g/dL)	12.01 ± 1.45	11.90 ± 1.80	12.00 ± 1.31	0.964
PLT (×1000/*μ*L)	331.86 ± 194.32	321.44 ± 91.17	289.82 ± 78.99	0.279
Vit.D (ng/mL)	12.15^b^ ± 3.43	20.63^a^ ± 7.35	23.41^a^ ± 8.06	0.023
Ca (mmol/L)	2.33 ± 0.16	2.28 ± 0.22	2.22 ± 0.31	0.526
Phos (mmol/L)	1.16^b^ ± 0.28	1.30^a^ ± 0.3	1.22^b^ ± 0.19	0.014
ALP (U/L)	98.50 ± 27.03	119.86 ± 85.05	113.87 ± 54.97	0.641
ESR (mm/hr)	69.71^a^ ± 16.79	48.52^ab^ ± 25.1	38.65^b^ ± 19.9	0.01
DAS28-ESR	5.34^a^ ± 0.29	4.01^b^ ± 0.52	2.76^c^ ± 0.27	<0.001

BMI: body mass index (18–25 kg/m^2^); WBC: white blood cells (4.5–11.0 ×1000/*μ*L); Hb: hemoglobin (12.0–16.0 g/dL); PLT: platelets (130–400 ×1000/*μ*L); Vit.D: vitamin D (sufficient >30 ng/mL), Ca: calcium (2.12–2.52 mmol/L); Phos: phosphorus (0.81–1.58 mmol/L); ALP: alkaline phosphatase (50–136 U/L); ESR: erythrocyte sedimentation rate (0–20 mm/hr); DAS28-ESR: disease activity score in 28 joints using erythrocyte sedimentation rate.

*P* value was determined according to ANOVA test.

Letters with different superscripts in the same row are significantly (*P* < 0.05) different according to post hoc LSD test.

**Table 3:** Vitamin D and DAS28-ESR bivariate Pearson correlation (*r*) with other independent variables (*n* = 102).

Independent variable	Vit.D *r* (*P* value)	DAS28-ESR *r* (*P* value)
Age	−0.175 (0.061)	0.001 (0.991)
Wt	−0.196 (0.068)	0.178 (0.154)
Ht	−0.117 (0.287)	−0.007 (0.958)
BMI	−0.140 (0.204)	0.177 (0.169)
Vit.D	1	−0.277 (0.014)
ESR	−0.034 (0.734)	0.453 (<0.001)
DAS28-ESR	−0.277 (0.014)	1

BMI: body mass index; Vit.D: vitamin D; ESR: erythrocyte sedimentation rate; DAS28-ESR: disease activity score in 28 joints using erythrocyte sedimentation rate.
